# Health-related quality of life and related factors among elderly persons under different aged care models in Guangzhou, China: a cross-sectional study

**DOI:** 10.1007/s11136-019-02107-x

**Published:** 2019-01-16

**Authors:** Shu-Wen Su, Dong Wang

**Affiliations:** School of Health Services Management, Southern Medical University, 1023 Shatai Road, Guangzhou, 510515 China

**Keywords:** Elderly persons, HRQoL, Aged care, Community-based home care, Institutional care, China

## Abstract

**Purpose:**

To analyze health-related quality of life (HRQoL) and related factors among elderly persons receiving community-based home care and institutional care in Guangzhou, a large city of mainland China.

**Methods:**

A representative sample of 1600 subjects aged 60 years and over residing in communities and nursing homes was randomly selected through stratified sampling. The 12-item Short Form Health Survey version 2 (SF-12v2) was used to assess HRQoL.

**Results:**

In total, 1014 elderly persons under different aged care models responded to the survey (response rate 63.4%) and 1000 were eligible for data analyses. Compared with the elderly receiving community-based home care or private institutional care, those in public institutional care had the lowest scores on the physical component summary (PCS, 36.89 ± 10.44) and the mental component summary (MCS, 47.16 ± 11.14). Number of chronic diseases, loneliness, and age were the most common significant factors (*P* < 0.05) affecting PCS and MCS. The interaction term between aged care model and number of chronic diseases significantly affected PCS (*β* = − 0.165, *P* < 0.05), indicating a stronger association between these factors for participants receiving community-based home care than institutional care. The interaction term between aged care model and loneliness had a significant effect on MCS (*β* = 0.189, *P* < 0.05), indicating a weaker association between loneliness and MCS for participants receiving community-based home care.

**Conclusions:**

This study found poor HRQoL among the elderly in Guangzhou. The main factors associated with the physical and mental HRQoL of elderly persons included number of chronic diseases, loneliness, age, and education level. It also revealed the moderating effects of aged care model on HRQoL, suggesting specific health management strategies for elderly in community-based home care and institutional care, respectively.

## Introduction

Population aging has become an increasingly serious social problem in the world. According to the “World Report on Ageing and Health” by the World Health Organization (WHO), the proportion of older persons in many countries (including China) will reach over 30% by 2050 [1]. The process of population aging in China is much faster than in many low-, middle-, and high-income countries. Moreover, by 2050, China is expected to have the largest group of citizens aged 80 years and over in the world, amounting to 90.4 million persons [2]. Guangzhou, a large-sized Chinese city, faces the same problem, as its residents aged 60 years and over account for 17.27% of the population [3], which exceeds the national average. However, little information is available regarding the general health status of elderly persons living in Guangzhou.

There are various types of long-term care for elderly persons. In general, three main categories exist, namely, informal home care (provided at home by family members), community-based formal home care (skilled and semi-skilled home care services at home), and nursing home care (institutional long-term care services in nursing home) [4]. With the dramatic aging of Chinese population, shrinking family scale [5], empty nest phenomenon [6], and “getting old before getting rich” [7] becoming increasingly more prevalent, the health of Chinese elderly has been closely monitored, as traditional informal home care has become costly and short-handed. Based on the above reasons, improving community-based home care and institutional care is imperative. Thus, this study focused on two of the above aged care models (community-based home care and institutional care) and furtherly defined the following concepts [8]. Community-based home care involves elderly still living in their own homes and communities but receiving social care services such as living care, medical rehabilitation, and spiritual comfort provided by the community. Institutional care means that elderly persons live in social organizations or institutions where comprehensive services such as nursing, accommodation, and living care are provided. According to the ownership of institutions, nursing homes are mainly divided into public and private institutions.

Health status, quality of life (QoL), and health-related quality of life (HRQoL) are three concepts closely related [9, 10]. According to the WHO, being healthy involves having good physical and mental health, and social well-being, and not merely the absence of disease or infirmity [11]. However, traditional health assessment indicators (such as life expectancy, potential years of life lost, average years of life lost, disability-free life expectancy) can only reflect one aspect of health, and it is difficult to meet the standards of the multidimensional concept of health. The WHO defined QoL as individuals’ perception of their living conditions associated with their goals, expectations, standards, and concerns in the context of their culture and value systems [12]. HRQoL was derived from the perspective of clinical medicine and health care [13] to evaluate the effects of health on QoL. Although HRQoL has been extensively studied in community-dwelling [14, 15] and institutionalized elderly [16, 17], it has seldom been assessed simultaneously in both populations [18], which is also the case for factors associated with HRQoL.

As we know, HRQoL may be affected by many factors. Two recent studies both in Nanjing and Jinzhou, a provincial capital of Southeast China and a medium-size city of Northeast China, respectively, indicated that the HRQoL of community-dwelling older persons was affected by age, education level, marital status, and chronic diseases, among other factors [10, 19, 20]. However, other important factors such as aged care model, loneliness, and number of chronic diseases [21–24] should also be taken into consideration.

In addition, due to differences in economy, culture, and geography, the conclusions obtained based on the populations of Nanjing and Jinzhou cannot be transferred to the Guangzhou population. Guangzhou is deeply influenced by foreign social culture and to a certain extent is representative of developed cities in China. At the same time, Guangzhou’s economy is prosperous, which allows it the possibility to catch up with the aged care models in developed countries. Therefore, studying the HRQoL and associating factors of the elderly in Guangzhou will contribute to improving the aged care models in developed regions of China.

The information available on the HRQoL of elderly persons living in Guangzhou is limited. Thus, this study aimed to address this gap by analyzing the HRQoL in this population and investigated the impact of different factors on HRQoL under different aged care models, aiming to provide references for local health care policy makers and public health researchers to design appropriate health management strategies for the elderly population.

## Methods

### Study population and data collection

This cross-sectional study recruited elderly men and women in six communities and two nursing homes in Guangzhou. Elderly persons participated voluntarily in our study; they were aged 60 years and over, according to the specifications of the WHO and the United Nations regarding old persons in developing countries [25], and had clear consciousness without cognitive or communicative impairments. Patients with dementia and psychosis were excluded from our study.

**Fig. 1:**
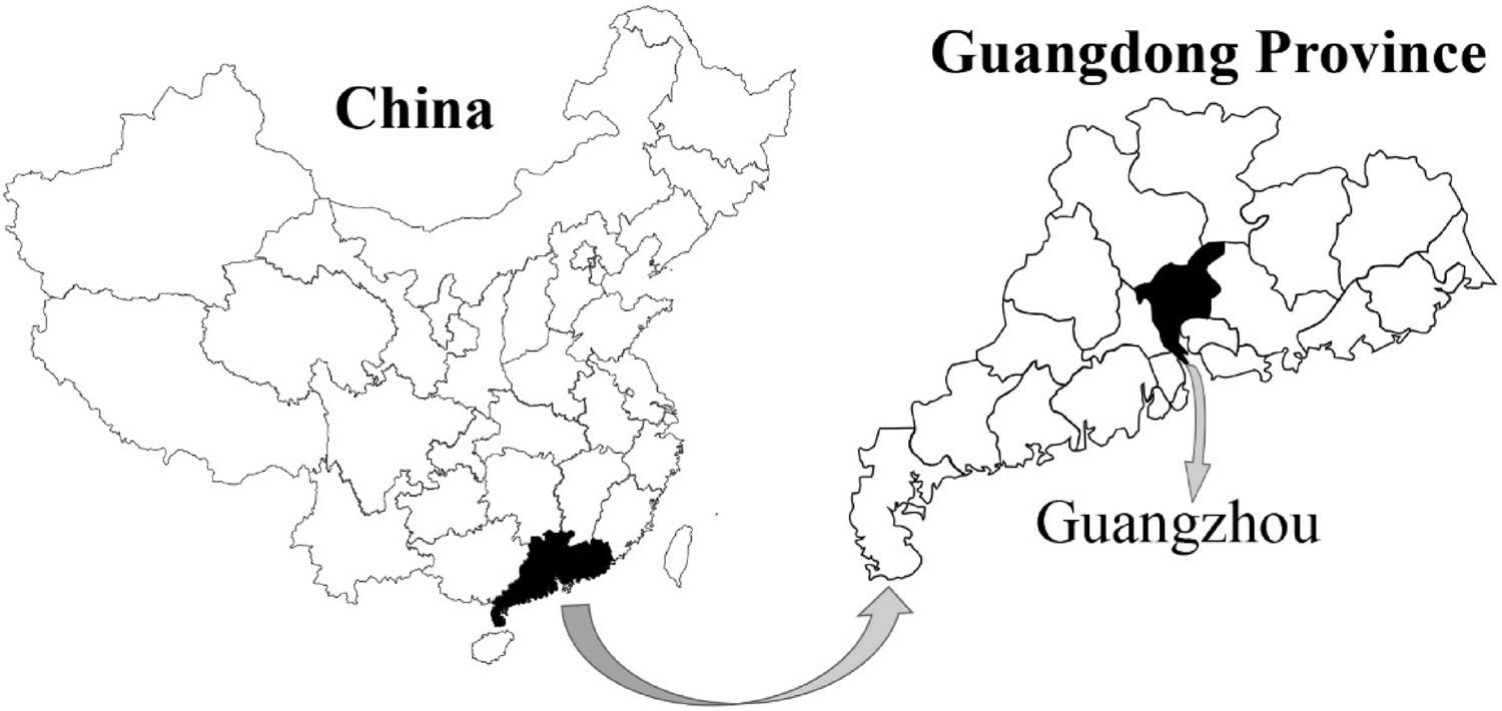
Geographical map of Guangzhou, Guangdong Province, China

Guangzhou is the provincial capital of Guangdong Province, South China. As shown in Fig. 1, it is located in south-central Guangdong and is divided into 11 districts, across which the distribution of the elderly population is uneven. On one hand, community-dwelling participants are dispersed across various communities and so, were sampled using a three-stage stratified sampling approach. First, according to Guangzhou’s elderly population distribution reported by the government in 2017, this study reclassified the 11 districts into four aging types: districts with more than 200,000 elderly persons, districts with 150,000–200,000 elderly persons, districts with 100,000–150,000 elderly persons, and districts with less than 100,000 elderly persons. Second, six representative districts were selected from the above four types in this study. Next, six communities were selected from each selected district. Subsequently, participants were randomly selected from the eligible candidates listed on the residents’ registration records provided by the local management agencies of community-based home care. A total of 800 questionnaires were distributed and 456 questionnaires were eventually collected.

On the other hand, the institutionalized elderly participants were sampled using a two-stage stratified sampling method, as they resided in nursing homes. First, according to ownership and control, the nursing homes in Guangzhou were divided into two types, namely, public and private institutions. Next, one large-scale (nursing beds ≥ 1000) public nursing home and one large-scale private nursing home were selected in this study. Subsequently, participants were randomly selected from the eligible candidates listed on the records of the nursing homes. A total of 800 questionnaires were distributed and 558 questionnaires were eventually collected.

### Measures

A questionnaire survey was conducted, which included general information and a HRQoL measurement scale (SF-12v2). General information included age, gender, educational level, marital status, aged care model, loneliness, number of chronic diseases. The SF-12v2 has been proven to be a practical, reliable, and valid scale for measuring HRQoL in both general and specific populations, and is available in multiple language translations, including Chinese [20, 26]. The SF-12v2 was derived from the 36-item Short Form Health Survey (SF-36), a well-recognized generic HRQoL measure, which was revised into a second version (SF-36v2) in 1996 [27]. However, the length of these tools limits their usefulness in some populations and large-scale studies. In order to improve usefulness, the SF-12 was first developed as a shorter version of the SF-36, and the SF-12v2 is its revised version [20]. The brevity of the SF-12v2 makes it an appealing tool to assess elderly persons’ HRQoL in large-scale studies. However, due to copyright issues, the application of the SF-12v2 in China is limited, and studies on HRQoL of elderly persons in mainland China using the SF-12v2 are still rare.

The SF-12v2 contains 12 items, and includes eight scale scores and two component summary scores. The eight subscales are scored from 0 to 100 (higher scores indicating better HRQoL), and include physical functioning (PF), role physical (RP), bodily pain (BP), general health (GH), vitality (VT), social functioning (SF), role emotional (RE), and mental health (MH) [28]. Physiological function, role physical, bodily pain, and general health are combined into the physical component summary (PCS), while vitality, social functions, role emotional, and mental health are combined into the mental component summary (MCS) [29]. Regarding the scoring of the SF-12v2, each of the eight subscales is scored following the standard scoring manual. The PCS and the MCS scores, ranging from 0 to 100 with a mean of 50 and standard deviation (SD) of 10, are calculated using the QualityMetric Health Outcomes™ Scoring Software 5.1 based on the US norm-based scoring algorithm. Permission to use the simplified Chinese SF-12v2 in this study was obtained from OptumInsight Life Sciences, Inc.

The Chinese SF-12v2 was administered to 50 elderly persons (23 men and 27 women) in a pilot study for the purpose of testing its reliability and validity. In the pilot study, Cronbach’s alpha reliability coefficient for all SF-12v2 items was 0.89. Moreover, convergent validity and discriminant validity were satisfactory for all SF-12v2 items. In addition, factor analysis indicated a two-factor structure (physical and mental health), with a cumulative variance contribution of 72.84%. In general, the above findings in the pilot study suggested that the SF-12v2 is a reliable and valid measure of HRQoL for elderly persons in our study.

### Quality control

The project leader organized recruitment, training, and grouping of investigators, and a group leader with strong communication skills and team experience was assigned to each survey site. The investigators, who were proficient in Mandarin and Cantonese, were systematically trained to be competent to administer the survey. The questionnaire was completed anonymously to ensure the privacy of the participants. Based on the consent of management staff of the local community organizations or nursing homes, the investigators introduced the purpose and principle of this survey to eligible elderly persons. After obtaining their informed consent, the elderly persons were taught how to fill in the questionnaires. In general, most participants completed the questionnaires independently. In special cases, the investigators assisted the elderly who had mobility issues, visual impairment, or illiteracy by reading items and filling in the questionnaires according to participants’ dictated answers. Missing or double answers were checked when the investigators collected the questionnaires. If such problems were found, the participants were asked to complete the information or make corrections at that point. Thus, the occurrence of missing or invalid answers was minimized, although not completely avoided. In this study, the questionnaires with missing data or double answers were considered invalid and excluded from the analysis.

### Statistical analysis

Data were managed using Epidata Version 3.0 in this study. Statistical analyses were carried out using Statistical Package for the Social Sciences (SPSS) version 20.0 (IBM SPSS Statistics for Windows, Version 20.0; IBM Corp., Armonk, New York, USA). Descriptive analysis for sociodemographic variables and HRQoL scores was performed using frequencies, percentages, means, and SDs. In order to preliminarily examine the associations between participants’ characteristics and HRQoL, univariate analyses including *t* test and one-way analysis of variance (ANOVA) were conducted. To further investigate the potential predictors of elderly persons’ HRQoL, a multiple stepwise linear regression analysis was performed with PCS and MCS scores as dependent variables, and variables significantly (*P* < 0.05) associated with the PCS/MCS scores in univariate analyses as independent variables. In the multiple stepwise linear regression analysis, variables associated with the PCS/MCS scores with *P* < 0.05 were entered, and those with *P* > 0.1 were removed. In addition, to investigate whether the predictors of HRQoL were the same in both community-based home care and institutional care groups, multiple linear regression using the enter method was performed by adding interaction terms between care model and other predictors. Two-sided *P* < 0.05 was considered significant in all analyses.

## Results

### Sociodemographic characteristics

In total, 1014 urban elderly persons under different aged care models responded to the survey (response rate 63.4%). Among them, 1000 valid questionnaires were included in the analysis, while 14 invalid questionnaires were deleted because of incomplete data or double answers. The age range of participants was 60–108 years, and the mean age was 78.34 (SD 9.38). Frequency distribution of sociodemographic characteristics is shown in Table 1.

**Table 1:** Frequency distribution of sociodemographic characteristics (*N* = 1000)

Factors	Group	*N*	%
Gender	Male	332	33.20
	Female	668	66.80
Age (years)	60–69	215	21.50
	70–79	244	24.40
	80–89	440	44.00
	≥ 90	101	10.10
Aged care model	Institutional care	549	54.90
	Public institutional care	379	37.9
	Private institutional care	170	17.0
	Community-based home care	451	45.10
Marital status	Married	403	40.30
	Separated	34	3.40
	Widowed	498	49.80
	Divorced	14	1.40
	Single	51	5.10
Education level	Illiteracy	149	14.90
	Elementary school	267	26.70
	Middle school	186	18.60
	High school/secondary	182	18.20
	Associate degree education or more	216	21.60
Loneliness	Never/rarely feel lonely	597	59.70
	Sometimes/often/always feel lonely	403	40.30
Number of chronic diseases	0	72	7.20
	1–2	493	49.30
	3–4	335	33.50
	≥ 5	100	10.00

### HRQoL

Table 2 shows the scores on HRQoL. The mean PCS score was 39.92 (SD 10.33) and the mean MCS score was 49.08 (SD 9.85). Moreover, compared with participants receiving community-based home care or private institutional care, the elderly receiving public institutional care had lowest scores on PCS (36.89 ± 10.44) and MCS (47.16 ± 11.14). Among the mean scores on the eight subscales, GH (36.65 ± 25.51) was the lowest, and MH (70.06 ± 20.89) the highest.

**Table 2:** Health-related quality of life of elderly people in Guangzhou (*N* = 1000)

Scales	Aged care model	Scores (mean ± SD)				
		Total	Age group			
			60–69 (*N* = 215)	70–79 (*N* = 244)	80–89 (*N* = 440)	≥ 90 (*N* = 101)
PCS		39.92 ± 10.33	44.99 ± 9.38	40.97 ± 10.21	37.78 ± 10.02	35.93 ± 9.52
	1	37.17 ± 10.18	37.85 ± 9.16	38.92 ± 11.30	37.04 ± 10.01	34.93 ± 9.50
	1.1	36.89 ± 10.44	36.78 ± 9.47	37.49 ± 11.63	37.21 ± 10.30	35.23 ± 10.01
	1.2	37.80 ± 9.57	42.52 ± 6.10	42.22 ± 9.89	36.75 ± 9.51	33.42 ± 6.47
	2	43.27 ± 9.51	46.77 ± 8.57	42.55 ± 9.00	39.77 ± 9.80	39.33 ± 8.97
MCS		49.08 ± 9.85	49.12 ± 8.65	49.31 ± 10.08	49.20 ± 9.81	47.94 ± 11.72
	1	47.85 ± 10.79	45.61 ± 11.26	46.94 ± 10.93	48.50 ± 10.30	47.67 ± 12.19
	1.1	47.16 ± 11.14	45.24 ± 11.69	46.91 ± 11.78	47.78 ± 10.45	46.54 ± 12.29
	1.2	49.39 ± 9.81	47.23 ± 9.65	46.98 ± 8.84	49.76 ± 9.95	53.36 ± 10.34
	2	50.58 ± 8.33	50.00 ± 7.66	51.14 ± 8.99	51.13 ± 8.07	48.84 ± 10.16
PF		48.60 ± 39.57	67.91 ± 36.00	55.64 ± 39.17	40.06 ± 37.46	27.72 ± 36.04
RP		51.13 ± 30.74	61.57 ± 29.21	55.17 ± 32.39	46.88 ± 28.76	37.62 ± 29.87
BP		62.85 ± 26.96	68.95 ± 24.85	62.19 ± 26.69	60.34 ± 27.64	62.38 ± 27.30
GH		36.65 ± 25.51	43.02 ± 26.59	34.47 ± 24.13	34.94 ± 25.15	35.74 ± 26.14
VT		58.60 ± 27.12	66.51 ± 21.77	60.25 ± 26.79	55.51 ± 28.18	51.24 ± 29.45
SF		59.88 ± 32.29	70.93 ± 28.46	65.47 ± 31.41	54.32 ± 32.30	47.03 ± 32.65
RE		64.45 ± 26.14	67.97 ± 24.60	65.11 ± 26.98	63.49 ± 25.57	59.53 ± 28.89
MH		70.06 ± 20.89	68.72 ± 18.79	70.34 ± 20.99	71.02 ± 21.12	68.07 ± 23.75

Aged care model (1 = institutional care, 1.1 = public institutional care, 1.2 = private institutional care; 2 = community-based home care)

### Predictors of HRQoL

Multiple linear regressions were performed to explore the predictors of physical and mental HRQoL in elderly persons. PCS (or MCS) score was used as the dependent variable, and the significant sociodemographic factors (including aged care model, loneliness, age, number of chronic diseases, education level, and marital status) in univariate analysis (*P* < 0.05) were covariates. We first assessed relationship between aged care model and HRQoL. In the second step we included the other covariates, and in the last step we added the interaction term between aged care model and loneliness, that between aged care model and age, and that between aged care model and number of chronic diseases.

As shown in Table 3, for the full sample of elderly persons, we found that the effect of aged care model on PCS was significant (*P* < 0.05) in Model 1 and Model 2. However, the aged care model did not significantly affect PCS (*β* = 0.405, *P* > 0.05) (Table 3, Model 3). In addition, the effect of number of chronic diseases on PCS was also significant (*P* < 0.05) in Model 2 and Model 3, though the effect in Model 3 (*β* = − 0.259) became weaker than that in Model 2 (*β* = − 0.328) when the interaction terms were included. The interaction term between aged care model and number of chronic diseases was significant (*β* = − 0.165, *P* < 0.05) in Model 3, suggesting the effect of number of chronic diseases on PCS was associated with the aged care model. Number of chronic diseases, loneliness, age, education level, and the interaction term between aged care model and number of chronic diseases were factors significantly affecting PCS in Model 3 (*P* < 0.05). The significant negative interaction indicated that the relationship between number of chronic diseases and PCS was stronger for participants receiving community-based home care than for those receiving institutional care.

**Table 3:** Relationships between related factors and PCS score (multivariate analysis, *N* = 1000)

Independent variables	Model 1^b^		Model 2^c^		Model 3^d^	
	*B* (*β*^a^)	SE	*B* (*β*)	SE	*B* (*β*)	SE
Aged care model	6.101*** (0.294)	0.628	2.070** (0.100)	0.644	8.410 (0.405)	5.320
Loneliness	–	–	− 4.315*** (− 0.205)	0.584	− 4.916*** (− 0.233)	0.760
Age	–	–	− 0.146*** (− 0.133)	0.034	− 0.120* (− 0.109)	0.049
Number of chronic diseases	–	–	− 2.241*** (− 0.328)	0.198	− 1.773*** (− 0.259)	0.247
Education level	–	–	0.456* (0.061)	0.206	0.430* (0.057)	0.205
Marital status	–	–	–	–	–	–
Interactions						
Aged care model × loneliness					1.928 (0.066)	1.190
Aged care model × age					− 0.053 (− 0.189)	0.069
Aged care model × number of chronic diseases					− 1.295* (− 0.165)	0.408
*F*	94.396		75.928		49.635	
*R* ^2^	0.086		0.276		0.286	
Adj *R*^2^	0.085		0.273		0.280	
Δ*R*^2^	0.086***		0.190***		0.010***	

(1) Multiple linear regression analysis. (2) PCS score in Short Form-12 v2 was used as the dependent variable. (3) Independent variables were entered as categorical variables, except for age and number of chronic diseases which were entered as continuous variables. Aged care model (0: institutional care, 1: community-based home care); Loneliness (0: never/rarely feel lonely, 1: sometimes/often/always feel lonely); Education level (1: illiteracy, 2: elementary school, 3: middle school, 4: high school/secondary, 5: associate degree education or more); Marital status (0: widowed/divorced/single; 1: married/separated). (4) Adjusted for the relevant variables in Table 1. (5) ^a^Numbers in parentheses are standardized coefficients. (6) *P < 0.05; **P < 0.01; ***P < 0.001

^b^In Model 1 (multiple enter linear regression analysis), “aged care model” was entered in the equation

^c^In Model 2 (multiple stepwise linear regression analysis.), “aged care model” and other covariates were included in the equation

^d^In Model 3 (multiple enter linear regression analysis), the covariates significant in Step 2 and three interaction terms (“Aged care model × Loneliness,” “Aged care model × Age,” and “Aged care model × Number of chronic diseases”) were entered in the equation

The predictors of mental HRQoL are shown in Table 4. In Model 3, loneliness (*β* = − 0.591), number of chronic diseases (*β* = − 0.107), and the interaction term between aged care model and loneliness (*β* = 0.189) were significant factors (*P* < 0.05) affecting MCS. The significant positive interaction suggested that the effect of loneliness on MCS was associated with the aged care model. The effect of loneliness on MCS was stronger for participants receiving institutional care than for those receiving community-based home care.

**Table 4:** Relationships between related factors and MCS score (multivariate analysis, *N* = 1000)

Independent variables	Model 1^b^		Model 2^c^		Model 3^d^	
	*B* (*β*^a^)	SE	*B* (*β*)	SE	*B* (*β*)	SE
Aged care model	2.731*** (0.138)	0.620	1.481* (0.075)	0.619	− 8.506 (− 0.430)	5.078
Loneliness	–	–	− 9.584*** (− 0.478)	0.555	− 11.858*** (− 0.591)	0.718
Age	–	–	0.109*** (0.104)	0.033	− 0.030 (− 0.029)	0.047
Number of chronic diseases	–	–	− 0.819*** (− 0.126)	0.190	− 0.694* (− 0.107)	0.235
Education level	–	–	–	–	–	–
Marital status	–	–	–	–	–	–
Interactions						
Aged care model × loneliness					5.300*** (0.189)	1.134
Aged care model × age					0.112 (0.422)	0.066
Aged care model × number of chronic diseases					− 0.442 (− 0.059)	0.390
*F*	19.396		89.183		55.901	
*R* ^2^	0.019		0.264		0.283	
Adj *R*^2^	0.018		0.261		0.278	
Δ*R*^2^	0.019***		0.245***		0.019***	

(1) Multiple linear regression analysis. (2) MCS score in Short Form-12 v2 was used as the dependent variable. (3) Independent variables were entered as categorical variables, except for age and number of chronic diseases which were entered as continuous variables. Aged care model (0: institutional care, 1: community-based home care); Loneliness (0: never/rarely feel lonely, 1: sometimes/often/always feel lonely); Education level (1: illiteracy, 2: elementary school, 3: middle school, 4: high school/secondary, 5: associate degree education or more); Marital status (0: widowed/divorced/single; 1: married/separated). (4) Adjusted for the relevant variables in Table 1. (5) ^a^Numbers in parentheses are standardized coefficients. (6) *P < 0.05; **P < 0.01; ***P < 0.001

^b^In Model 1 (multiple enter linear regression analysis), “aged care model” was entered in the equation

^c^In Model 2 (multiple stepwise linear regression analysis.), “aged care model” and other covariates were included in the equation

^d^In Model 3 (multiple enter linear regression analysis),the covariates significant in Step 2 and three interaction terms (“Aged care model × Loneliness,” “Aged care model × Age,” and “Aged care model × Number of chronic diseases”) were entered in the equation

**Fig. 2:**
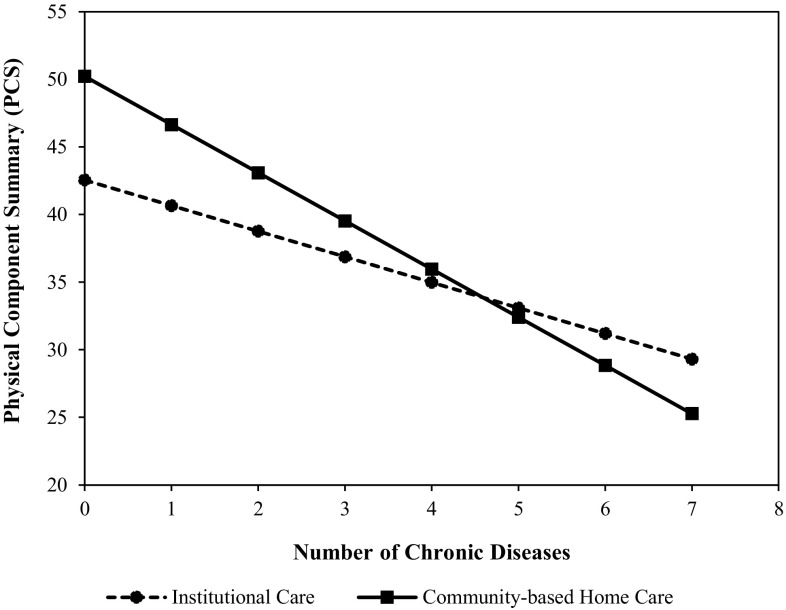
The effect of number of chronic diseases on PCS is associated with the aged care model

**Fig. 3:**
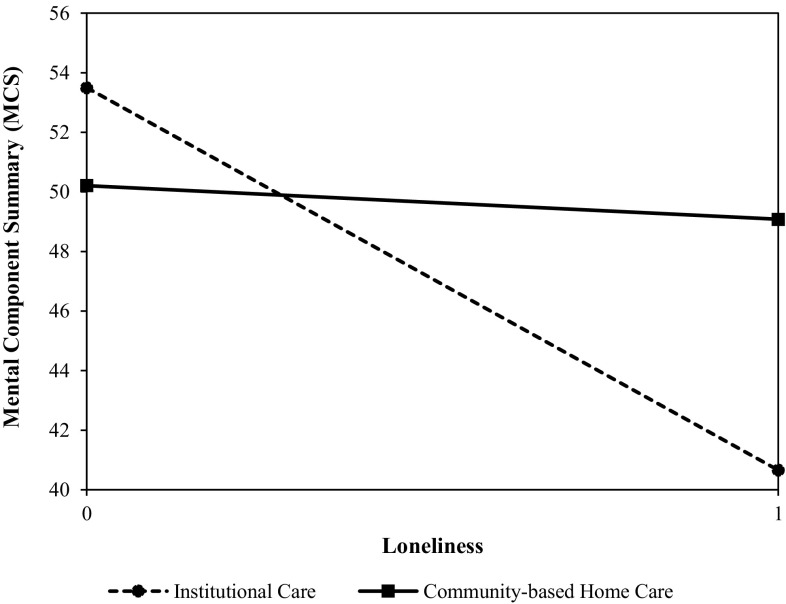
The effect of loneliness on MCS is associated with the aged care model. Loneliness (0: never/rarely feel lonely, 1: sometimes/often/always feel lonely)

In order to examine the differential effect of number of chronic diseases on PCS for the two groups (community-based home care and institutional care), we conducted simple estimates analysis (see Fig. 2). The results showed that indeed the effect was stronger for the community-based home care group than for the institutional care group (*b* = − 3.563, *P* < 0.001; *b* = − 1.892, *P* < 0.001, respectively). Figure 3 showed that the effect of loneliness on MCS was weaker for the community-based home care group (*b* = − 6.448, *P* < 0.001; institutional care group: *b* = − 11.958, *P* < 0.001).

## Discussion

This study provided a report on the HRQoL of elderly persons receiving different types of aged care in Guangzhou, China. The mean scores of PCS and MCS obtained in this study were lower than those of elderly persons in similar studies in Brazil [30] and the USA [31], but higher than those reported for elderly persons in Iran [32]. Thus, the Chinese elderly persons in this study showed medium HRQoL compared to those in some upper middle-income countries; however, there was a certain gap between the HRQoL in this study and that in a high-income country.

The community-based home care group scored higher than the institutional care group on the two component summaries. Furthermore, we also found that the private institutional care group scored higher than the public institutional care group on both PCS and MCS. This may be partly explained by the general health status and living conditions of these populations. On one hand, in the community-based home care group, there is a large portion of elderly persons with low-level need for care, while many elderly persons in the institutional group have high need for care. On the other hand, compared with the public institution group, the private institution group could receive better living care, more skilled nursing, etc.

The main factors associated with the physical and mental HRQoL of elderly persons included number of chronic diseases, loneliness, age, and education level. As the number of chronic diseases increased, the HRQoL of the elderly deteriorated, under both aged care models. This is consistent with previous studies showing that having a chronic disease could reduce HRQoL significantly and living with multiple chronic diseases could reduce HRQoL even further [33, 34]. Second, a negative correlation was found between loneliness and HRQoL among elderly under different aged care models. Previous studies have shown that loneliness was an important reason for the poor self-rated health of elderly persons [35, 36] and strongly related to elderly depression [37]. Compared with elderly participants who never or rarely felt lonely, the lonely elderly participants scored lower on physical and mental HRQoL. However, the causal mechanism underlying the association between loneliness and HRQoL is still unclear in this study. The opposite relationship, in which lower HRQoL leads to loneliness, is also possible, as lower HRQoL may lead to loneliness via difficulties in maintaining social relationships [38]. Third, with increasing age, the physical HRQoL of the elderly persons showed a downward trend. Previous studies have demonstrated that aging was related to the deterioration of self-rated health, which is mainly reflected in low physical HRQoL [20, 39, 40]. However, this study showed that aging had a positive impact on the mental HRQoL of the community-dwelling participants, which is different from previous research [15]. This unexpected finding was not statistically significant, but may indicate that the living environment of community-based home care is more beneficial to the mental health of elderly persons than that of institutional care. Fourth, higher educational level had a positive impact on the physical HRQoL of participants. Similar studies have shown that elderly persons with a higher education level have better physical HRQoL than those with lower education [19, 41]. Fifth, marital status was not a significant factor associated with physical or mental HRQoL, which is inconsistent with previous studies [15, 19, 42]. It may be that, compared with other significant factors that were simultaneously included, this factor only affected HRQoL in a weaker way.

It is not surprising to find that number of chronic diseases, loneliness, age, and educational level were factors associated with elderly persons’ HRQoL, which is consistent with previous studies. However, this study also found that the above factors had different effects on HRQoL in community-based home care and institutional care groups. This study offers a unique perspective on the effects of aged care models on HRQoL, examining the moderating role of aged care model. On one hand, community-based home care can be considered as a risk factor, aggravating the deteriorating effects of chronic diseases on physical HRQoL. However, on the other hand, community-based home care can be considered as a protective factor, buffering the adverse effects of loneliness on mental HRQoL.

The number of chronic diseases had a higher negative impact on the physical HRQoL of the elderly receiving community-based home care relative to those receiving institutional care. This finding may reflect that the quantity and quality of medical services available to community-dwelling elderly persons cannot be guaranteed, and might be inferior to those received in institutional care. Since the nursing homes in this study were equipped with professional geriatric hospitals, institutionalized elderly persons can enjoy basic chronic disease management and professional treatment for illness or injury. In contrast, the elderly persons receiving community-based home care had a distinct disadvantage in accessing medical services. On the one hand, due to the unreasonable allocation of health resources in China, it is more difficult for community-dwelling elderly persons to access the professional medical services provided by the geriatric hospital or geriatrics department than it is for institutionalized elderly persons. On the other hand, the community health service departments have limitations in terms of health education and chronic disease management. Self-medication and self-treatment are still common among community-dwelling elderly persons, which may have made it more difficult to effectively control chronic diseases in the community-dwelling group.

Loneliness had a lower negative association on the mental HRQoL of the elderly receiving community-based home care than those in institutional care. This finding may be partly explained by the living environment in community-based home care, which may weaken the impact of loneliness on mental HRQoL. First, as the majority of institutionalized elderly persons in China live in double or triple rooms with other roommates, differences in physical health, daily routine, and lifestyle often make it difficult for residents to adapt to each other. Second, due to the lag in the development of the aged care industry in China, the number of caregivers in many nursing homes is insufficient [5] and the quality of care is low [43, 44]. Third, the institutionalized elderly persons not only lose their original social network, as nursing homes are often far from urban areas, but also often fail to integrate into new social networks, as they are generally not active enough to establish new social relations in nursing homes [45]. Fourth, compared with institutional care, community-based home care can meet elderly persons’ expectation of enjoying home joys to a greater extent, which is consistent with the traditional Chinese culture [44].

This study used a relatively large sample of elderly persons from communities and nursing homes in Guangzhou, a large city in mainland China. As known, Guangzhou has become a multicultural metropolitan city and migration from the whole country to Guangzhou is high and vibrant. Thus, our findings may be generalizable to other Chinese subgroups in economically developed regions of China (Eastern coastal cities of China, etc.) due to vast economic and cultural similarities.

In summary, the findings from our study could provide some references for policy makers and researchers to design appropriate health management strategies for elderly persons in community-based home care and institutional care.

With regard to community-based home care, three suggestions are provided as follows. Firstly, primary health care doctors should not be limited to providing medical services only in hospitals, but should also offer on-call medical services to elderly persons. We must give equal attention to these two service patterns in community health services. Elderly persons often have difficulties in walking; thus, their demand for on-call medical services is very high. Community health service providers should attend to the health needs of elderly persons and provide them with multi-faceted medical services. Secondly, on the basis of establishing health records, primary health care providers should provide regular follow-ups, health education, and disease prevention guidance for the elderly in the community. Thirdly, in order to help suitable elderly patients to stay in their community for as long as possible, the government should improve the policies on community-based home care (including long-term care insurance, community caregiver training), foster voluntary service organizations, and enhance informal neighborhood mutual aid networks.

As for institutional care, we provide three suggestions as follows. First, before implementing interventions, institutional care providers should fully understand the experience of loneliness among institutionalized elderly persons and grasp the key associating factors. On this basis, a precise intervention plan for loneliness can be developed, which mainly includes improving social skills, enhancing social support, increasing opportunities for social interaction, and changing negative social perceptions. Furthermore, in order to help institutionalized elderly persons to adapt to collective life, social support should be increased and an atmosphere of trust should be built in nursing homes. Thus, efforts need to be made in the following areas: (1) strengthening the professional training of caregivers; (2) improving the level of institutional management; and (3) supporting institutionalized elderly persons to actively participate in learning, physical exercise, and related leisure activities. In addition, in caring for institutionalized elderly persons, it is important to not only comply with doctors’ advice but also pay attention to humanistic concerns and provide psychological and social support. Only by comprehensively considering various factors (including physical and mental condition, living habits, hobbies) can we customize integrated care plans to improve the HRQoL of elderly persons.

## Limitations

Firstly, the study design was cross-sectional; thus, it was difficult to establish long-term causal relationships between elderly persons’ HRQoL and their sociodemographic characteristics. In the future, a longitudinal study design is needed to further investigate and clarify causal links. Secondly, the participants in this study were elderly persons aged 60 years or over, and some of them had a low education level or poor understanding ability, which might have made it difficult for them to fully understand the questionnaire. Thirdly, due to limited resources, this study did not include lifestyle factors that may affect HRQoL, such as smoking, drinking alcohol, and physical exercise. Despite these limitations, the results of this analysis provided a large picture of the HRQoL of elderly persons in Guangzhou and initially explored some important factors closely related to the health of this population, which may facilitate further research using a prospective study design.

## Conclusions

In summary, this study found poor HRQoL among elderly persons in Guangzhou, a large city in mainland China. The performance of the questionnaire was satisfactory and provided a representative picture of elderly persons’ HRQoL status in Guangzhou, China. The elderly persons receiving community-based home care generally showed better HRQoL than those receiving institutional care. Furthermore, we also found that those in private institutional care scored higher than those in public institutional care on both physical and mental HRQoL.

This study also showed that sociodemographic factors such as number of chronic diseases, loneliness, age, and education level affected elderly persons’ HRQoL and offered a unique perspective on the effects of aged care models on HRQoL, examining their moderating role. The effects of loneliness and aged care model on elderly persons’ HRQoL deserve attention, as once their effects on HRQoL have been further verified, it will be wise to improve HRQoL by reducing loneliness and improving the aged care model, which might be more forward-looking and economic than disease interventions alone. This study not only provides a comparative reference for the application of the SF-12v2 in different elderly populations, but also suggests some health management strategies for elderly persons in community-based home care and institutional care, respectively. These findings will help local governments to design a preliminary intervention framework for promoting the HRQoL of elderly persons.
